# Recruiting former melanoma patients via hospitals in comparison to office-based dermatologists in a register-based cohort study that required indirect contact

**DOI:** 10.1186/s12874-017-0425-2

**Published:** 2017-11-22

**Authors:** S. R. Zeissig, V. Weyer-Elberich, K. Emrich, H. Binder, S. Fischbeck, B. H. Imruck, P. Friedrich-Mai, M. E. Beutel, M. Blettner

**Affiliations:** 1 Cancer Registry of Rhineland-Palatinate, Grosse Bleiche 46, 55116 Mainz, Germany; 2 Institute of Medical Biostatistics, Epidemiology and Informatics (IMBEI), University Medical Center of the Johannes Gutenberg-University Mainz, Mainz, Germany; 3 Department of Psychosomatic Medicine and Psychotherapy, University Medical Center of the Johannes Gutenberg-University Mainz, Mainz, Germany; 4 Department of Psychosomatic Medicine and Psychotherapy, Medical Psychology and Medical Sociology, University Medical Center of the Johannes Gutenberg-University Mainz, Mainz, Germany

**Keywords:** Registry-based study, Response proportions, Non-responder analysis, Recruitment procedure

## Abstract

**Background:**

There are detailed reviews about different recruitment strategies, but not with regard to differences between recruitment of hospital-based versus office-based physicians. Within this study, the two different recruitment schemes are compared. Advantages and disadvantages of different ways of recruitment in registry-based studies are discussed.

**Methods:**

In a cross-sectional cancer-registry-based study, long-term melanoma patients were contacted by dermatologists rather than directly by the registry on the basis of the legal situation. Logistic regression models and generalized estimating equations were used to assess effects of various patient and physician characteristics on participation and data quality. Especially differences between hospital-based versus office-based dermatologists are evaluated.

**Results:**

Seventy two out of 112 contacted dermatologists took part in the study (64.3%). The cooperation proportion was 52.2% (689 participants/1320 contacted patients). Participants and non-participants differed regarding age and sex, but not regarding other social demographic factors and cancer stage.

We did not observe a difference in patient participation between hospital-based versus office-based dermatologists (OR 1.08 [CI 0.84–1.39]; *p* = 0.57). However, medical data provided by the cancer registry were better for participants registered and recruited by hospitals.

**Conclusions:**

In cohort studies with epidemiological cancer registries, recruitment via physicians has potential disadvantages and is more complex. If this indirect way of contact is mandatory, we recommend recruitment procedures including hospital-based rather than office-based physicians. However, physician characteristics were not associated with outcome.

## Background

Registry-based studies have the advantage of accessing representative samples of patients in a certain catchment area. However, patient contact may not be direct and instead be mediated by treating physicians, depending on the specific regulations underlying the registry of interest. In the study area (Rhineland-Palatinate, 4 million inhabitants), direct access to patients is prohibited by law for data protection reasons. Physicians who have reported their patients to the cancer registry must be contacted and motivated to send letters to their former patients. To provide insights into potential benefits (and also downsides) of such an access scheme, we report results of a cross-sectional study on long-term melanoma patients (MeLa study) who were recruited in cooperation with the Rhineland-Palatinate Cancer Registry [1].

The primary objectives of the MeLa study were to assess the psychosocial burden of long-term melanoma survivors and their psychosocial care needs (quality of life and distress as primary endpoints) as well as to identify psychosocial care determinants (sociodemographic, medical, psychological) as reported elsewhere [1, 2]; however, the focus in this article is on investigating the indirect access scheme to give more general recommendations for study designs.

Gurwitz et al. found that recruitment procedures involving the treating physician as an active gatekeeper may limit access to patient participation in studies from the perspectives of both researchers and potential study participants [3]. For example, patients may feel pressure to participate if their physician has sent them a letter [4]. Thus, lower response proportions compared to other registry-based studies could be anticipated. On the other hand patients feeling pressured to participate react in different ways: Some of them may take part despite that, or actually because of it, others may refuse participation. In addition, it could be an advantage that in this study the treating physician, as a person of trust, has asked patients to participate [5].

It is known that systematic nonresponse can bias inferences that are made about the population. The nonresponse rate cannot serve as the proxy for nonresponse bias. Davern postulates that paradata (data collected about the survey operations, for instance, contact histories) and a nonresponse bias analysis should be produced and published for every survey if the results are published in a scientific journal [6].

Therefore, in this paper we describe recruitment procedures of a cancer-registry-based survey and investigate whether responders and non-responders differ in terms of personal characteristics and course of disease. Differences between the physicians (hospital or office-based; working in a rural or an urban setting) who were asked to take part in the study are described as well as the corresponding patient groups. We examine whether there are different effects on the participation proportion or data quality between the patients who were contacted by hospitals versus those contacted by practitioners. The advantages and disadvantages of this indirect manner of contacting study participants are discussed, and recommendations for further studies with similar recruitment procedures are presented.

## Methods

### Study population

All physicians in Rhineland-Palatinate are legally bound to report incident cancer cases to the confidential office of the cancer registry. The incoming reports are checked for completeness and plausibility, the tumor data are coded and the personally identifiable data are encrypted (asymmetric RSA) by the cancer registry. To contact cancer patients for this survey, decryption of personally identifiable data is only possible after permission from the ethics committee and the Ministry of Health.

The estimated completeness of incident melanoma notifications in Rhineland-Palatinate is more than 95% [7]. Initially all melanoma patients who had survived at least 5 years after a diagnosis between 2000 and 2005 and were at least 14 years old at the time of diagnosis were selected (*n* = 2371). Further 290 patients were excluded because they were not informed about the report to the cancer registry. Patients who had not been reported by a dermatologist were then excluded (*n* = 259). The inclusion/exclusion criteria for study participants are shown in Table 1.

**Table 1:** Criteria of inclusion and exclusion of study participants

Inclusion criteria	Exclusion criteria
Surviving patients with malignant melanoma at least 5 years after diagnosis	Patient died
Patients are reported by dermatologists to the cancer registry Rhineland-Palatinate (year of diagnosis 2000–2005)	Patients reported to the cancer registry only by other physicians than dermatologists
At least 14 years old at time of diagnosis	Not able to understand the purpose of the study, e.g. because of lack of language skills or mental disabilities
Signed informed consent	Patients who were not informed about the report to the cancer registry

### Recruitment procedures

Due to the legal situation for the cancer registry in Rhineland Palatinate, only dermatologists who had originally registered the patient were allowed to contact the patient. If more than one physician had reported the same patient to the registry, we decided to contact the hospital in-patient dermatological departments first. The reporting physician received the letter for the potential study participant from the study center. The physician checked and updated the address of the former patient, informed the study center about possible reasons for non-participation of the patient (e.g. deceased, mentally disabled) and sent the signed letter back to the confidential office of the cancer registry. If the documents were not returned after 6 weeks, the physician received a reminder. If there was no response after another 6 weeks, the physician was contacted via phone up to two times (first and second phone-in session). If the physician agreed to take part in the study, documents were sent to him/her again if necessary. If he/she refused or was unavailable after the 2nd phone-in session, no further contact was attempted.

Study information, informed consent, questionnaires and the signed form letter of the physician were sent to the study participants by the study center. They were offered access to a study assistant by phone. Responses were checked by the study center. Potential study participants who did not react to the letter of interest (1st patient contact) within 6 weeks were contacted once again (2nd patient contact). For this the study center required the cooperation of the physician once more. The procedure was repeated once more, and non-responders were not contacted further. Patients who will willing to participate signed the informed consent document, including agreeing to the data protection protocol, filled out the questionnaire and sent the documents back to the study center. The returned questionnaires were checked for completeness. If the study participant had approved being contacted via phone by the study center, some questionnaires were completed by telephone if necessary. Questionnaires were pseudonymized with a study-ID and transmitted to the Department of Psychosomatic Medicine and Psychotherapy at the University Medical Center of the Johannes Gutenberg University Mainz. Study-related illness data (e.g. cancer stage or localization of the melanoma) were transmitted for data analysis by the cancer registry and matched by study-ID.

For checking the patients’ addresses and the other data mentioned above, the physician received an expense allowance at a rate of €30.00 / patient. For the patients who had to be contacted a second time after 6 weeks (second patient contact), the physician received another expense allowance at a rate of €10.00/ patient (renewed signature and sending back to the study center).

### Physician and patient characteristics and questionnaires

Information about tumor site, tumor stage, time since diagnosis and place of residence was derived from data in the cancer registry. Municipality keys from the German Bureau of Statistics (Gemeindekennziffern) were used to code for urban or rural place of residence and to describe the size of the catchment area of the physicians (the number of community codes the responding patients belong to for each dermatologist).

To code the UICC-stage, different editions of the TNM were used according to the year of diagnosis: Up to 2003, TNM 5th edition (German version, Springer Verlag 1997); for year of diagnosis 2004 or later, TNM 6th edition (German version, Springer Verlag 2002). Time since diagnosis was calculated for participants as the time between the date of diagnosis and the date of return of the questionnaire to the study center. Of course, the latter date did not exist for non-participants, so age at termination of recruitment (05_15_2012) served as a proxy.

Depression, generalized anxiety disorder, Quality of Life, Fatigue and other emotional and functional disorders were measured using a rash of standardized psychometric tests incorporated in a questionnaire of 20 pages [8–14].

### Statistical analysis

Response proportions were calculated according to the method presented by Slattery et al. [14] with slight modifications (regarding the two-step approach of recruitment). *P*-values calculated to detect differences between participants and non-participants and early−/late-participants were not corrected for multiple testing.

Recruitment proportions for different groups of dermatologists (hospital vs. medical practice) were calculated. Logistic regression was used to assess the potential effects on the participation of physicians (Table 2; model 1) and patients. (Table 2; model 2). As a further important indicator of effects of the access scheme under investigation, we considered the missing values in study questionnaires, which might be indicative of data quality. Specifically, we considered the potential effects of various patient and physician characteristics on whether a participant had any missing values (yes/no) in the study questionnaires. For answering this question we performed generalized estimating equations with a logic link and with an unstructured correlation structure for accounting the correlation from patients with the same physician (Table 2; model 3).

**Table 2:** Logistic regression and generalized estimating equation models

Model	Outcome variable	Predictor variables
Model 1	Participation of physician: yes/no	*Physician characteristics:* Location of hospital/practice (urban/rural), size of catchment area (number of community codes the responding patients of each dermatologist belong to), median age of all patients assigned to the physician, median tumor stage of all patients assigned to the physician
Model 2	Participation of patient: yes/no	*Patient characteristics:* hospital-based physician: yes/no, age, sex, UICC (stage), place of residence (urban/rural)
Model 3	Missing values in study questionnaire: yes/no	*Patient characteristics:* Age, sex, early−/late-responder, time since diagnosis, social stratum, place of residence (urban/rural), recruited by hospital/office-based physician
		*Physician characteristics:* Hospital/practice, location of hospital/practice (urban/rural), size of catchment area (number of community codes the responding patients of each dermatologist belong to), median age of all patients assigned to the physician, median tumor stage of all patients assigned to the physician, recruitment proportion, number of contacts before participation

All statistical analyses were conducted using SAS version 9.4 (SAS Institute, Cary, NC).

## Results

### Participation of dermatologists

**Fig. 1:**
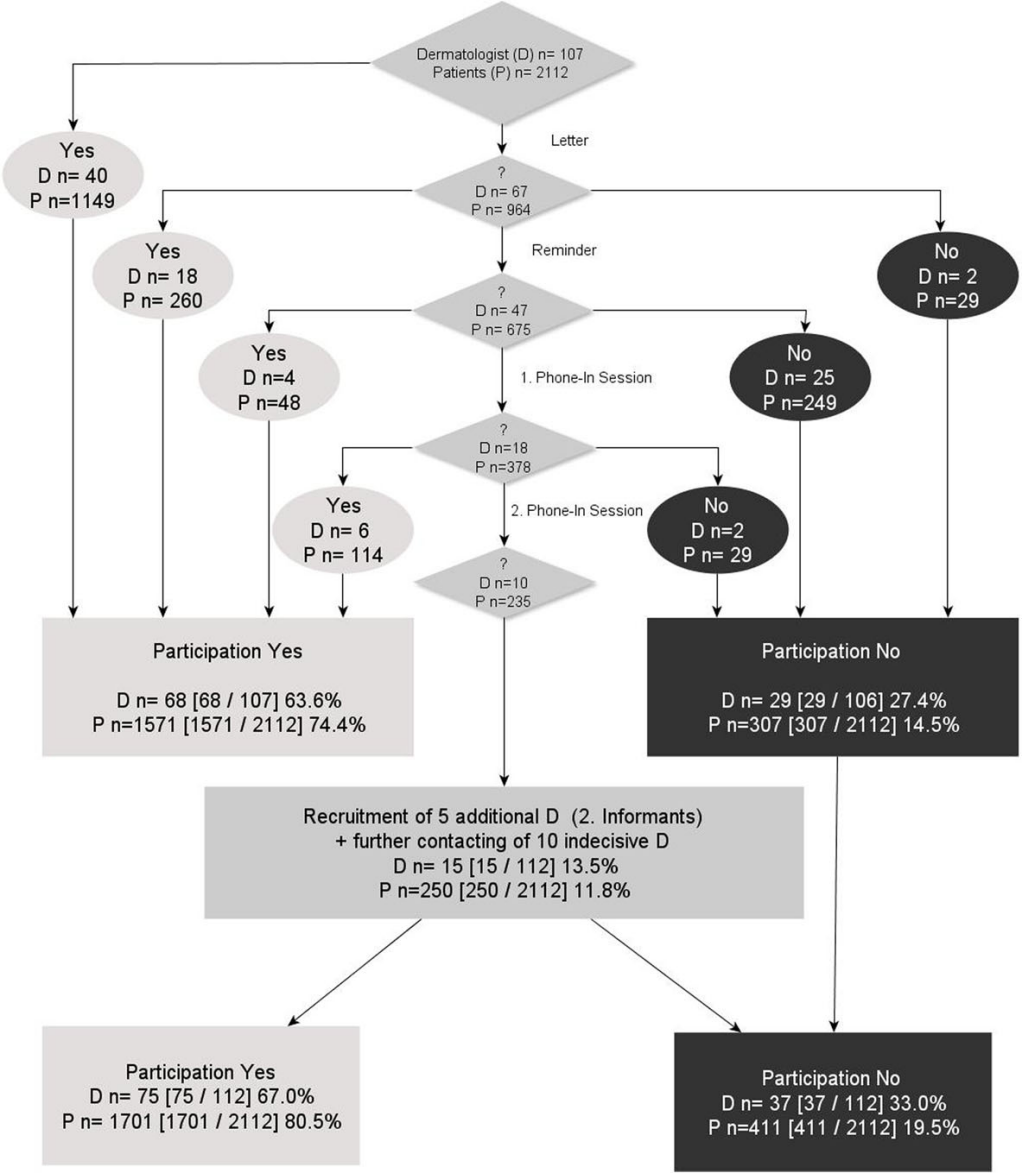
Recruitment of dermatologists (22.7.11–10.5.12)

In the present paper we consider institutions (*n* = 72), counting dermatologists in group practices as one dermatological institution in three cases, whereas the previous publication looked at single physicians (*n* = 75) rather than at institutions. After completion of the recruitment phase (May 2012), 69 dermatological practices (63.3% of invited practices) and 3 hospitals (100% of invited hospitals) out of 112 institutions contacted had agreed to take part in the study (overall participation rate 64.3%). Figure 1 indicates the corresponding numbers of patients as given in our previous publications regarding 75 individual dermatologists [1, 2].

In 67 of the 112 cases, a postal reminder had to be sent. Forty seven dermatologists were contacted in a first phone-in session and 18 medical practices in a second phone-in session, when they were then requested to finally confirm or refuse study participation. On this occasion physicians were asked for the reasons for non-participation. Reasons for physician non-participation (*n* = 40) were “no interest in any kinds of studies or particularly in studies on quality of life”, “lack of time”, “too much bureaucracy”, “no personal contact with the patient anymore” or giving up their medical office. There was a time delay of 4 month between the first contact with dermatologists and the first contact with patients.

Larger size of catchment area corresponds to an increase of physician participation (Model 1; OR 1.09 [CI 1.02–1.17]; *p* = 0.02). This effect also remains if only office-based dermatologists are considered (excluding hospitals): OR 1.08 [CI 1.01–1.17]; *p* = 0.03. Age (OR 0.99 [CI 0.93–1.05]; *p* = 0.71) or cancer stage (OR 1.58 [CI 0.44–9.59]; *p* = 0.53) of eligible patients does not show effects on the recruitment of the dermatologists.

### Participation of patients

**Fig. 2:**
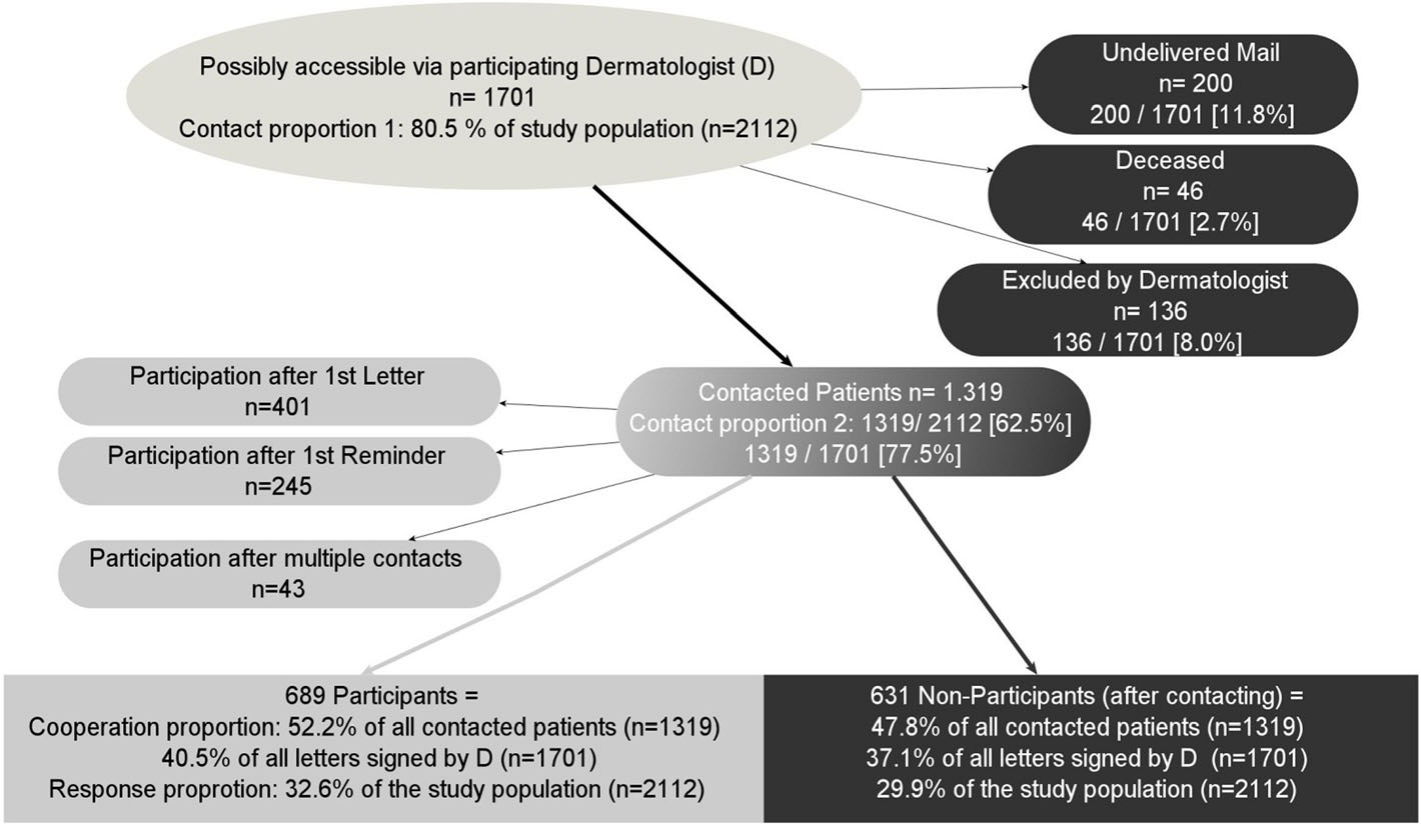
Recruitment of patients

Initially, 2112 patients who met the inclusion criteria were registered in the cancer registry; of these, any patients who were not “able to understand the purpose of the study” were excluded by the physicians or the patients themselves during the process of recruitment. Only 1701 individuals were potentially accessible at the beginning of recruitment; this is because only physicians who took part in the study could contact patients. For data protection reasons, the cancer registry was not allowed to research addresses for patients when mail was returned as undeliverable (*n* = 200). In 46 cases, physicians or relatives reported back that the individual was meanwhile deceased, and another 136 patients were excluded by their dermatologist because of dementia or other causes for being unable to take part in the study. Ultimately, 1319 people were contacted successfully. 689 (52.2%) of them signed written consent and completed the study questionnaire, most of them after receiving the first letter of invitation (*n* = 401) and were therefore classified as “early responders” (Fig.2; Table 3). All other participants who completed the study questionnaire after the first reminder or later (single patients responded first after seven contacts) were classified as “late responders” (Table 3).

**Table 3:** Comparison between study participants and non-participants in terms of patients’ social demographics and disease characteristics

	Participants (*n* = 689)						Non-participants (*n* = 1012)^c^		*p*-value^4^
	Early responder (*n* = 401)^a^		Late responder (*n* = 288)^b^		All participants (*n* = 689)				
	n	%	n	%	n	%	n	%	
Sex									0.03
Male	197	49.1	138	47.9	335	48.6	438	43.3	
Female	204	50.9	150	52.1	354	51.4	574	56.7	
Cancer stage at time of diagnosis (UICC) (Register data)^d^									0.15
Stage I	205	51.1	160	55.6	365	53.0	492	48.6	
Stage II	17	4.2	17	5.9	34	4.9	68	6.7	
Stage III	4	1.0	3	1.0	7	1.0	16	1.6	
Stage IV	0	0	0	0	0	0	3	0.3	
unknown	175	43.6	108	37.5	283	41.1	433	42.8	
Localization at time of diagnosis (ICD-10) (Register data)									0.13
C43.6 + C43.7: Extremities	193	48.1	136	47.2	329	47.8	502	49.6	
C43.2-C43.4: Head−/ Neck-Area	50	12.5	43	14.9	93	13.5	168	16.6	
C43.5: Trunk	148	36.9	100	34.7	248	36.0	317	31.3	
C43.8+ C43.9: Unspecified/Overlapping Areas	10	2.5	9	3.1	19	2.8	25	2.5	
Place of residence									0.08
Urban	289	72.1	207	71.9	496	72.0	687	67.9	
Rural	112	27.9	81	28.1	193	28.0	325	32.1	
Age at end of recruitment (years)^e^									< 0.0001
Median	62.0		63.0		62.0		69.0		
Lower Quartile	50.0		50.0		50.0		51.0		
Upper Quartile	73.0		74.5		74.0		79.0		
Time since diagnosis (years)^f^									0.18
Median	9.0		9.0		9.0		9.0		
Lower Quartile	7.0		8.0		8.0		8.0		
Upper Quartile	11.0		10.0		10.0		10.0		

^4^Comparison of participants and non-participants: Chi^2−^Test and Fisher’s Exact Test (categorical variables); Median Test (continuous variables)

^a^Participation after first mail-out, percentages refer to all participants (*n* = 689)

^b^Up to 7 reminders after first mail-out, percentages refer to all participants (*n* = 689)

^c^Total register-based sample: *n* = 2112, percentages refer to all non-participants (*n* = 1012)

^d^UICC-Stage: For year of diagnosis up to 2003 according to TNM 5th edition (German version, Springer Verlag 1997), for year of diagnosis 2004 or later according to TNM 6th edition (German version, Springer Verlag 2002); Subdivisions according to TNM 6 in A or B were summarized in respective stages

^e^Age at termination of recruitment (15.05.2012): Difference between date of birth and end of recruitment

^f^Period of time between date of diagnosis by month and termination of recruitment

Response proportions depending on different study populations (subsets of the 2112 identified patients) following a slightly modified version of the method introduced by Slattery et al. [15] were

- *Contact proportion 1* (Percentage of patients for whom there was a theoretical chance of contacting via the participating physician): 1701/2112 = 80.5%
- *Contact proportion 2* (Percentage of patients ultimately contacted via the participating physician): 1319/2112 = 62.5%
- *Cooperation proportion* (Percentage of patients who participated from all those who were contacted): 689/1319 = 52.2%
- *Response proportion* (Percentage of patients who participated from the total number of patients selected): 689/2112 = 32.6%

### Description of participants and non-participants

There were no statistically significant differences between participants and non-participants (patients for whom there had been a theoretical chance of being contacted via their treating dermatologist, but who finally did not take part in the study; *n* = 1012) regarding tumor site, cancer stage, time since diagnosis and place of residence (urban/rural), but there were slight differences regarding the age distribution and sex in the univariate analysis: Non-participants were older and more often female (Table 3).

**Fig. 3:**
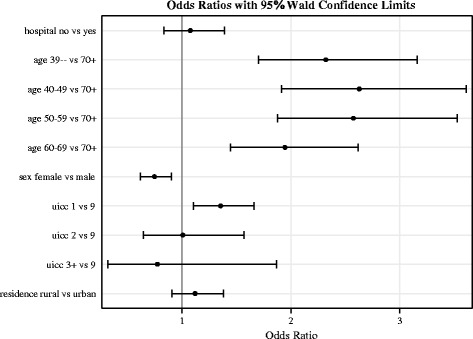
Odds Ratios and Confidence intervals for patient participation (Model 2). Description of Fig. 3: UICC 3 + = UICC 3 and UICC 4 tumor stage, UICC 9 = unknown tumor stage

Results of the multivariate analysis of patient participation (Model 2) confirm these findings as presented in Fig. 3: Odds ratios and confidence intervals show that a larger proportion of males (compared to females) participated in the study (OR 0.75 [CI 0.62–0.91]) and that more patients with an earlier stage (UICC = 1) participated than patients with unknown stage (OR 1.36 [CI 1.11–1.66]). A smaller proportion of older than younger patients participated (e.g. age 60–69 versus 70+: OR 1.95 [CI 1.45–2.62]). No effect was shown for place of residence (rural versus urban residence: OR 1.12 [CI 0.91–1.38]).

One hundred and thirty one participants were recruited by hospitals and 558 by medical practices. The recruitment rates for medical practices varied widely, spanning the rates observed for hospitals. We did not observe a difference in patient participation between hospital-based versus office-based dermatologists (Model 2; OR 1.08 [CI 0.84–1.39]; *p* = 0.57).

The frequency of contacts until the questionnaire was sent back to the study center was recorded. Four hundred and one participants (58.2%) sent back all study documents after the first mail-out and were therefore classified as “early responders” (Table 3). All other participants (*n* = 288; 41.8%) were classified as “late responders”, without differentiating whether they took part after one (*n* = 245; 35.6%), two (*n* = 29; 4.2%), three (*n* = 9; 1.3%), four (*n* = 4; 0.6%) or seven (*n* = 1; 0.2%) contacts (postal mailings or telephone calls). No relevant differences considering age, sex, cancer stage, time since diagnosis, localization of melanoma or place of residence were found between early and late responders (Table 3).

However, a larger number of participant contact attempts (≥ 3) corresponded to a greater likelihood of having missing values in questionnaires (Model 3; OR 2.55 [1.45–4.50]; *p*-value 0.0012). The same applies for elderly patients (OR 1.02 [1.01–1.04]; *p*-value < 0.0010) and female sex (OR 1.37 [1.01–1.86]; *p*-value 0.04) (Table 4).

**Table 4:** Missing values in study questionnaire (Model 3)

Effect	OR	95% Confidence Limits		*p*-value
Physician characteristics				
Location of hospital/practice (urban versus rural)	1.37	0.85	2.20	0.20
Median age of patients^a^ assigned to institution	0.99	0.97	1.02	0.72
Size of catchment area^a^	1.01	0.10	1.02	0.10
Median tumor stage of all patients assigned to the physician	0.97	0.56	1.67	0.91
Number of contacts before participation	1.14	0.89	1.46	0.29
Recruitment proportion	0.67	0.15	2.91	0.59
Patient characteristics				
Age of participant (years)	1.02	1.01	1.04	0.001
Sex (female versus male)	1.37	1.01	1.86	0.04
Participation after 2 contacts^b^	1.25	0.90	1.73	0.19
Participation after ≥ 3 contacts^b^	2.55	1.45	4.51	0.001
Time since diagnosis (years)	0.98	0.88	1.08	0.64
Place of residence (urban versus rural)	0.93	0.67	1.27	0.64
Social stratum (middle versus low)	0.66	0.48	0.91	0.01
Social stratum (high versus low)	0.69	0.46	1.03	0.07
Recruited by hospital versus office-based physician	0.50	0.20	1.26	0.14

^a^number of community codes the responding patients of each physician belong to

^b^Reference: Early responders (Participation after the first mail-out of study documents))

Regarding data quality, medical data provided by the cancer registry were better for participants recruited by hospitals than for those recruited by practices, but there was no statistically significant difference between the existence of missing values in the study questionnaires of hospital recruits versus participants recruited by medical practices (OR 0.50 [0.20–1.26]; *p*-value 0.14) (Table 4).

## Discussion

The aim of the current paper was to investigate potential effects of recruitment procedures in a registry-based study (the MeLa study) where initially no direct contact between the cancer registry or the research institutions involved and the eligible study participants was allowed.

Hence, we expected a lower participation rate compared to other registry-based studies. However, reporting of response proportions in epidemiological studies is not standardized, and comparisons across studies are often difficult because details identifying who is included are often lacking [16]. When we compare the response in the MeLa study to participation rates in similar registry-based cohort studies [17–21], we find different results depending on the manner of recruitment.

The highest cooperation proportion can be seen in a follow-up assessment of an existing cohort of longtime breast cancer survivors. Questionnaires assessing quality of life were sent to participants who had given permission to be re-contacted (*n* = 238). One hundred and eighty two out of these participated (Cooperation proportion: 76.5%) [17].This finding is in line with recruitment results in a study with long-term melanoma survivors and population controls in Minnesota. Participants from a previously conducted case-control study were recruited for a cross-sectional survey and 62.0% of those cases also completed the follow-up survey [18]. In cooperation with the Hamburg Cancer Registry, a set of self-reporting questionnaires were mailed to breast cancer survivors. This direct approach resulted in a cooperation proportion of 66.3% (1083/1633) [19]. Another legal situation and other contact formalities exist at the Cancer Registry of Schleswig-Holstein, where only 762 persons out of 1503 registered former melanoma patients meeting inclusion criteria could be contacted because the other 741 melanoma survivors were registered only with a pseudonym (no name or postal address). For this reason the contact proportion was only 50.7% (762/1503). However, the cooperation response was 59.1% because 450 out of the 762 contacted patients took part in the study [20]. Another population-based study was set up to examine the quality of life in long-term survivors of breast, colon and prostate cancer. Cancer registries of the German federal states of Schleswig-Holstein, North Rhine-Westphalia, Rhineland-Palatinate, Hamburg, Bremen and Saarland cooperated in this study. This means different legal situations, which led to different recruitment methods in the regions. Additionally, the cohorts from Saarland and Schleswig-Holstein described above were included. The overall cooperation proportion was 43.4%, as only 7162 patients out of 16,500 contacted patients completed the questionnaire [21].

Although the cooperation proportion of 52.2% in the MeLa study, despite the indirect access, is comparable to the similar registry-based studies described above, many potential study participants (*n* = 411; 19.5% of the whole study population) were never contacted because their physician did not take part in the study. We can only speculate about explanations for the result showing that with growing size of his or her catchment area, participation of the physician becomes more likely. Perhaps more potentially eligible patients justify the effort to participate for the office-based dermatologist and expense allowances seem to be more profitable.

Some patients could not be contacted because they were excluded by the dermatologist (*n* = 136; 6.4% of the whole study population). In the study of Vogel et al. 1.5% (17 out of 1.167) previously surveyed long-term melanoma survivors were excluded, because their physician denied re-contacting [18]. Therefore, the reporting physician’s own perceptions regarding a constellation of medical and/or psychosocial characteristics, as well as personal biases, determines whether approval is given to contact a patient to participate in the study [3]. As this could result in a selection bias, we did an indirect non-responder analysis. We did not find statistically significant differences between participants and non-participants regarding localization of the tumor, time since diagnosis and urban or rural place of residence. Non-participants more often belonged to the age group of 70+ years. Perhaps they had difficulties in understanding the study purposes or filling in the study questionnaire, or were less interested in taking part in the study. We do not have an explanation as to why non-participants in the MeLa study were more often female. In summary, we conclude that these findings support the assumption that pre-selection of patients by their treating physicians does not result in a severe bias.

Many researchers have published their experience or reviews of incentives and disincentives for research participation of office-based physicians [22–26]. They have identified several barriers, such as for example engaging a practice receptionist to relay the study information to the physician who ultimately decides about participation [22]. Lack of time is a barrier that could be overcome by transferring as much as possible of the study burden from participating physicians to project staff [23]. On the other hand, there are some facilitators described in the literature for the recruitment of office-based physicians: close liaison with local medical organizations and prominent members of the medical community, on-going personal contact with the practices, direct financial or indirect incentives (e.g. feedback on patients’ health outcomes), and recognition of the value of the practicing physician’s time [22, 24, 25]. In the MeLa study, some of these criteria were implemented: practitioners only had to sign prepared letters, check addresses and the possibility of participation of their patients and then send the letters back in a prepared and postpaid envelope. They also received expense allowances. Furthermore, the head of the Rhineland-Palatinate Association of Dermatologists (Berufsverband der Deutschen Dermatologen e.V., Landesverband Rheinland-Pfalz) recommended participation to its members in an email sent some weeks before recruitment started.

It could be an advantage in this study that the treating physician who asked patients for participation was a person of trust. Schwartz et al. showed that patients of physicians who allowed their names to be listed on the letter of invitation were more likely to engage in each step of the recruitment process in a randomized controlled trial of two psychosocial interventions [5]. Furthermore, this indirect approach to study participants has the advantage that the treating physician could identify individuals who are mentally or physically not able to participate or had recently died; relatives were not unnecessarily stressed from receiving requests for these patients to participate. Physicians probably could provide information on the new address of patients after their removal.

In contrast, there are some important disadvantages by indirectly contacting patients via the treating physicians: Financial efforts (expense allowances, postal charges), manpower requirements and a time delay until possible study participants can be contacted. The MeLa study also had to face each of these problems.

There are detailed reviews about different recruitment strategies, but not with regard to differences of recruitment of hospital-based versus office-based physicians [27, 28]. We assume that recruitment via clinics could be interpreted as a direct contact, because patients often do not know the name of the signing physician, and therefore they may have the impression that a large unknown institution – comparable to any other research institution – is contacting them. It is not surprising that the quality of medical data provided by the cancer registry was better for participants recruited by hospitals, because those institutions often have comprehensive tumor documentation systems. Yet, there was no difference in the probability of having missing values in the study questionnaires between participants recruited by medical practices versus hospital-based dermatologists. However, the more attempts were required to contact participants, the more frequently missing values showed up in the questionnaires. This is surprising because there is a bias in our data which works against this finding: There is no discrimination between different causes for multiple contacts to study participants, and in some cases questionnaires were completed in telephone interviews.

There is a substantial limitation of the current study. It is important to assess the potential causes of nonresponse, particularly when the response proportion is low [29]. However, we have information about non-participants only from aggregated and anonymized cancer registry data without specification of causes for nonparticipation (besides exclusion by the physician and undelivered mails).

## Conclusions

We could not show in our data that contacting eligible study participants via hospitals instead of office-based physicians can be expected to lead to higher participation rates.

However, if an indirect way of contact is mandatory, we recommend recruitment procedures including hospital-based rather than office-based physicians. We did not observe a distinct difference in recruitment rates, but our experience is that more patients could be reached with less effort when relying on hospital-based physicians. Moreover, the quality of medical information was higher for patients recruited by hospitals. For office-based physicians, we observed improved recruitment with a larger catchment area, which might also be relevant for other studies with indirect recruitment.

## Abbreviation

UICC: The Union for International Cancer Control
